# Evidence that CA3 is Underling the Comorbidity Between Pain and Depression and the Co-curation by Wu-Tou decoction in Neuropathic Pain

**DOI:** 10.1038/s41598-017-12184-y

**Published:** 2017-09-20

**Authors:** Chunyan Zhu, Qionghong Xu, Chao Wang, Zhiyun Mao, Na Lin

**Affiliations:** Institute of Chinese Materia Medica, China Academy of Chinese Medical Sciences, Beijing, 100700 China

## Abstract

In neuropathic pain (NP), the atrophy of hippocampus contributes to the comorbidity between pain, depression and the cognitive deficits. However, the exact mechanism underling the comorbidity, the effective control of the degenerations in hippocampus and the remission of the accompanied depressive symptoms are still lacking. Wu-Tou decoction (WTD) has been prescribed for inflammatory pain for thousands of years. In this study, we manifested the effects of WTD on the pain, depression and anxiety co-curative symptoms of NP. Moreover, we reported that WTD rescued the mal-regulated BDNF and TNF-α in hippocampal CA3 alone, which is proven contributing to the pain and induced psychiatric symptoms. Finally, analysis of biochemistry, morphology and electrophysiology exhibited the potential mechanism of WTD in CA3. We found that, in the late stage of SNL condition, WTD mediated the rescue of the down-regulated glutamate as well as its pre-synaptic vesicular glutamate transporters (VGLuT1) and the post-synaptic α-amino-3-hydroxy-5-methyl-4-isoxazolepropionic acid (AMPA) receptors in CA3. In sum, the targeted mediation of glutamatergic system in CA3 suggest that WTD may be responsible for the remission of the hypo-functioned CA3 glutamatergic neurons and further contribute to the co-curative effects of WTD.

## Introduction

Neuropathic pain (NP), triggered by the lesions to the somatosensory nervous system or the sustained activation of peripheral immune system, is a debilitating neurological condition of high clinical relevance (6.9 ~ 10%)^1,2^. Besides the heightened pain sensitivity, mood disorders such as depression and anxiety as well as the cognitive deficits have been frequently reported as the important syndromes of NP^3,4^. Therefore, for NP, the remission of the pain induced symptoms is as important as the alleviation of pain. Unfortunately, the effective treatment is still lacking. Pregabalin (PGB), most widely prescribed for NP and recommended by Food and Drug Administration (FDA) as first-line drug^5,6^, was found ineffective for pain modulated depression^7^. Other first-line drugs such as tricyclic antidepressant (TCA) and serotonin noradrenaline reuptake inhibitors (SNRIs) were also restricted to a minority of patients due to either serious side effects or the limited analgesic and anti-depression/anxiety effects^8^. To ameliorate this conundrum, increasing studies have been focused on the mechanism of co-morbidity between the heightened pain sensitivity and mood disorders. Traditional Chinese medicine formulas have been proven to be more favorable for the treatment of chronic pain^9^. Wu-Tou decoction (WTD), recorded in *Jin Gui Yao Lue* by Zhongjing Zhang in 3 century AD, is one of the most classic and effective formulas prescribed for joint disease and inflammatory pain. The prescription composition of WTD contains five herbs (Radix Aconiti, Herba Ephedrae, Radix Astragali, Raidix Paeoniae Alba and Radix Glycytthizae). By chemical profiling, 74 components including alkaloids, monoterpene glycosides, triterpene saponins, flavones and flavone glycosides are identified in the water extract of WTD and recognized responsible for the anti-inflammatory as well as the anti-oxidant activities^10,11^. In the complete Freund’s adjuvant-induced chronic inflammatory pain model, the effective ablation of the Ca^2+^channel dependent excessive transduction of peripheral nociceptive stimuli has been proven critical for the analgesic effects of WTD^12,13^. However, for NP, it is the maladaptive atrophy of brain nuclei, rather than the excessive activation of peripheral neurons, more important to the resistance and the complication of the disease^14^. In this study, for the first time, we evaluated the effects of WTD to the nociceptive, as well as the co-comorbidity symptoms of NP in the spinal cord ligation (SNL) mice. In addition, the co-curative effects of WTD further encouraged us to explore for the central mechanism and more emphasis was put on the brain nuclei.

Among the brain nuclei, the atrophy of hippocampus has been most widely reported and associated with the nociceptive behaviors, cognitive deficits as well as the depressive symptoms in pain conditions^15^. The most direct evidence of the chronic pain mediated pathological dysfunction of hippocampus came from the patients and animal models. Aggressive evidence of animal models suggested that hippocampus involved in nociceptive symptoms^16^ and associated mood disorders^17,18^ and cognitive dysfunctions^19^. In view of the important roles hippocampus assumed in the occurrence of the symptoms, the studies of the hippocampal neurons will provide more lights on the mechanism of co-comorbidity. Hippocampus is composed of three closely connected regions: hippocampus proper (CA1, CA3), dentate gyrus (DG) and subiculum^20^. Each component has been proven influenced by NP^15^. In acute stage, the injure induced nociceptive afferents facilitate the long-term potentiation in hippocampal CA3 and CA1 pyramidal neurons^21–23^. This immediate activation of hippocampus is crucial for the perception of pain and the making of evasive response^15^. On the contrary, the sustained pain signals have been proven harmful to the plasticity of hippocampal neurons^17,24,25^ and responsible for the development of cognitive as well as the depressive symptoms^17,18^. However, the long-term pathological consequences of hippocampal neurons and the association with the development of NP symptoms remain to be elucidated.

In the nerves system, brain-derived neurotrophic factor (BDNF) responses to the environment stimulus immediately and maintains both the transient and long-term plasticity of neurons. For NP, the hippocampal BDNF deficiency has been widely reported and recognized to be associated with the cognitive as well as depressive symptoms^26,27^. Therefore, the studies of BDNF signal pathway will further clarify the long-term pathological associations between hippocampal neurons and the co-occurrence syndromes. In addition, the excessive activation of immune systems has been widely reported in NP^28^. Among the elevated pro-inflammatory cytokines, the tumor necrosis factor alpha (TNF-α) has long been recognized as the linker between the abnormal activated immune system and the damaged nerves system^29^. Especially, in hippocampus, the pathological up-regulation of TNF-α has been proven responsible for the co-occurrence of the deficits in working memory^17^ and the depressive behaviors^18^ in NP animal models. In addition, the hippocampal injection of TNF-α has been proven inducible for the deficits of working memory^17^ and the persistent pain-like behaviors^30^. Therefore, both BDNF and TNF-α have been proven crucial for the co-morbidity. However, the mechanisms underlying the long-term deprivation of BDNF and up-regulation of TNF-α to hippocampal neurons have not been clearly clarified.

To clarify the pharmacological mechanism underling the long-term administration of WTD, we construed the spinal cord ligation (SNL) mice model and confirmed the WTD mediated remission of NP symptoms and the regulation of BDNF and TNF-α in CA3. On this basis, we hypothesized that: 1: The long term deprivation of BDNF and up-regulation of TNF-α in CA3 underline the co-comorbidity of the cognitive deficits, depressive, anxiety as well as the nociceptive behaviors; 2: The co-curative effects of WTD is dependent on CA3; 3. The WTD-mediated rescue of the morphological atrophy and hypofunction of CA3 glutamatergic neurons may underline the pharmacological mechanism. For this aim, mice injected with ANA-12 (antagonist of BDNF TrkB receptor) and TNF-α in CA3 was constructed to estimate the roles CA3 assumed in the long-term pathological and pharmacological processes. Further, to verify the WTD mediated rescue of hippocampal neurons, we performed the morphological analysis of CA3 pyramidal neurons *in vivo* and analysis of the activity-dependent plasticity of spines of hippocampal neurons *in vitro*. In addition, the quantifications of glutamate and its pre/post-synaptic receptors at the level of protein expression, the co-localization of BDNF and glutamate receptors by fluorescent double staining and the mEPSC analysis of hippocampal CA3 pyramidal neurons were further performed.

## Results

### WTD attenuates the SNL-induced mechanical allodynia, depressive and anxiety symptoms

**Figure 1:**
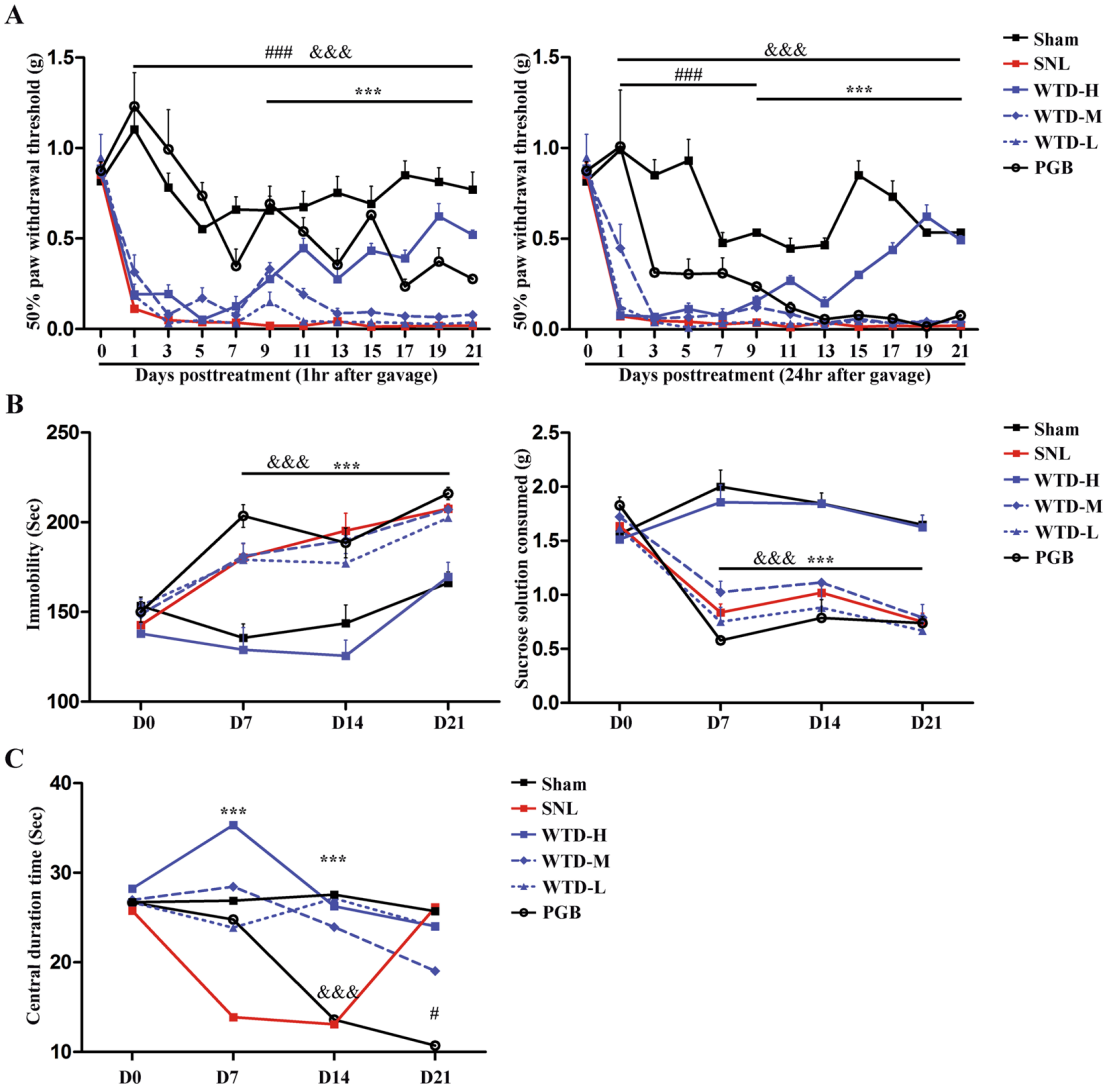
The behavior deficits in SNL mice and the revival by WTD. Grouped data (Mean ± S.E.M, n = 5–9 mice per group) show the alternations in pain (**A**), depression (**B**) and anxiety (**C**) behaviors at each time point. In Fig. 1A and B, ^&&&^denotes the significant (P < 0.001) difference between Sham and SNL. ^###^Denotes the significant (P < 0.001) difference between PGB and SNL. ***Denotes the significant (P < 0.001) difference between WTD-H and SNL. In Fig. 1C, ***denotes the significant (P < 0.001) difference between Sham/WTD-H/WTD-M/WTD-L and SNL. ^&&&^Denotes the significant (P < 0.001) difference between Sham/WTD-H/WTD-M/WTD-L and PGB. ^#^Denotes both the significant (P < 0.05) difference between PGB and Sham/SNL/WTD-H/WTD-M/WTD-L.

To evaluate the analgesic effects of WTD (high, medium and low doses), the nociceptive symptoms were estimated by the Von Frey filaments tests from D1 to D21 after the SNL operations. For SNL, the mechanical allodynia syndrome represented by the lower paw withdrawal thresholds maintained ranging from D1 to D21. For WTD, significant analgesic effects, shown as the increases of 50% paw withdrawal thresholds in the high dose group (WTD-H) detected at both 1 and 24 hrs after the daily administration, was detected from D9 to D21. For Pregabalin (PGB), increased threshold were manifested during the whole disease process exposure to the drug administration after 1 hour; whereas, the significant increases detected 24 hrs after PGB administration disappeared after D9, which indicates that the effectiveness of PGB is hampered by the long-term administration. In sum, compared to pregabalin, the effectiveness of WTD-H is more evident after the long-term administration. (Fig. 1A).

To evaluate the anti-depression effects of WTD, the depressive behaviors were assessed by the forced swimming and the sucrose consumption tests. For SNL, compared to sham, decreases of sucrose consumption and prolongations of immobility time in forced swimming tests was observed from D7 to D21, which suggests the development and maintenance of depressive symptoms during the following pathological stage. For WTD, compared to SNL, increases in sucrose consumption and the contraction of immobility time in forced swimming test was detected in each time point in WTD-H, rather than in other groups, which indicates the anti-depression effects of WTD-H. In addition, as reported by previous studies, no anti-depression effect of PGB was detected in our SNL models (Fig. 1B).

To evaluate the anti-anxiety effects of WTD, the anxiety behaviors were evaluated by the open field tests. For SNL, compared to sham, significant decrease of central duration time in the open field test was seen from D7 to D14, which suggests that the development and maintenance of pain-induced anxiety behaviors. For WTD, compared to SNL, the significant increases of central duration time were represented in high, medium and low dose groups on both D7 and D14. For Pregabalin, compared to SNL, the significant increases of central duration time was detected on D7 but disappeared on D14. Furthermore, compared to both sham and SNL, PGB showed decreased central duration time on D21. In sum, the effects of WTD on the SNL-induced anxiety were independent of doses. Although the short-term (consecutive 7 days) administration of PGB is effective for anxiety, it seems like the long-term (consecutive 14–21days) administration is ineffective and even inducible of anxiety (Fig. 1B).

In addition, analyzed by the Hematoxylin and Eosin staining, 21 days long-term application of WTD-H was proved harmless to the body weight, heart, liver and kidney (see supplementary Fig. S1). From this results, we confirm that long-term administration of WTD has low adverse effects.

### WTD selectively modulated the level of BDNF and TNF-α in CA3

Brain nuclei is crucial for moods coordination. The depression and anxiety-curative effects further indicate the brain-targeted mechanism of WTD (high dose). To further clarify the related brain regions, BDNF and TNF-α were quantified at protein level in brain tissues extracted on D7, 14 and 21 in the Sham, SNL and WTD groups (n = 9 mice from 3 independent batches of experiments).

**Figure 2:**
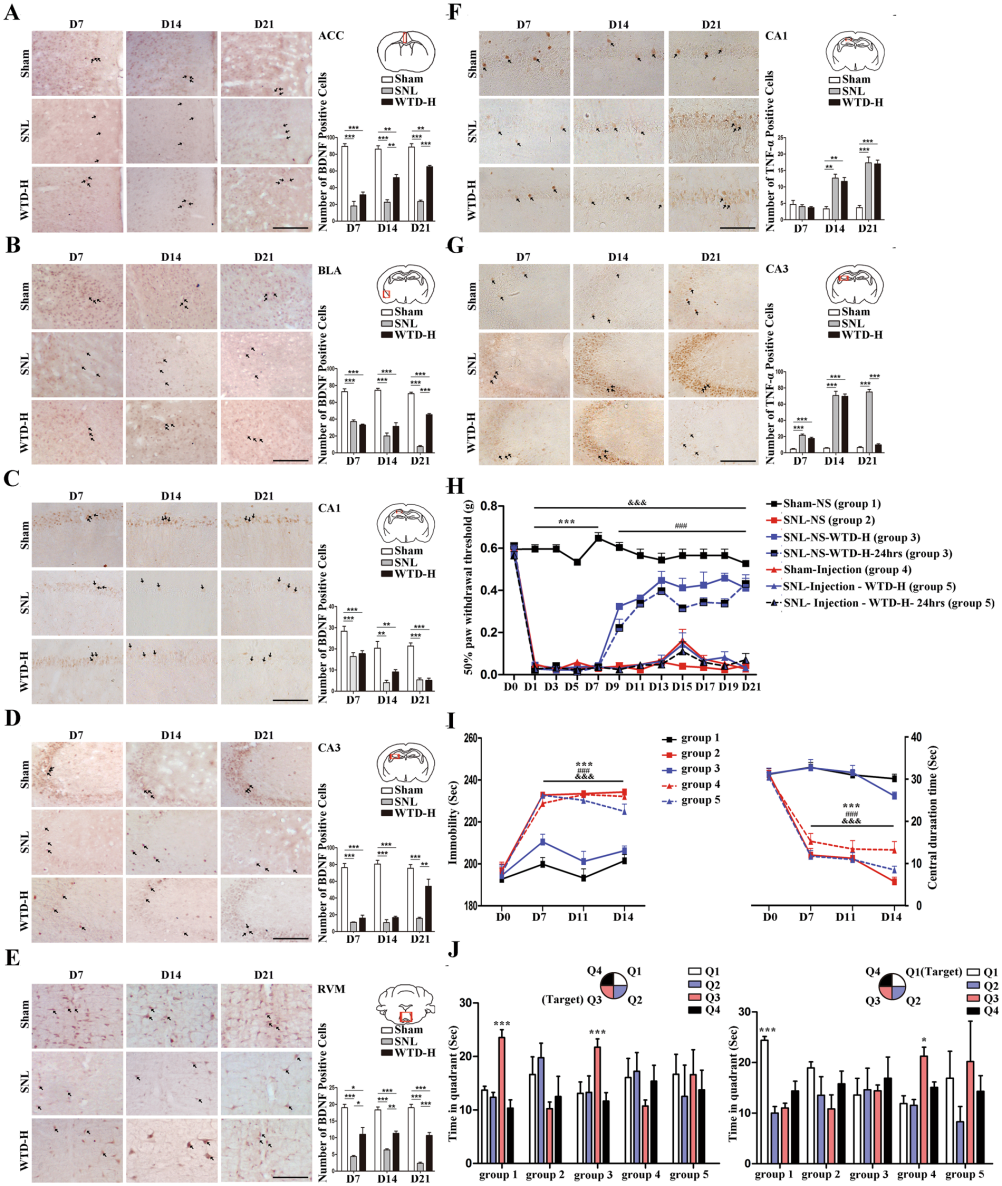
The mal-regulation of BDNF and TNF-α in SNL mice and the selective revival by WTD. The selectively regulation of BDNF in the anterior cingulate cortex (**A**), basolateral nucleus of amygdale (**B**), CA1 (**C**), CA3 (**D**) and rostroventromedial medulla (**E**) were quantified by IHC staining, scale bar 200 μm and analyzed in bar chart(Mean ± S.E.M, N = 9 mice) (*P < 0.05, **P < 0.01, ***P < 0.001). The selectively regulation of TNF- a in the hippocampus CA1 (**F**) and hippocampus CA3 (**G**) were quantified by IHC staining, scale bar 200 μm and analyzed in bar chart (Mean ± S.E.M, N = 9 mice) (*P < 0.05, **P < 0.01, ***P < 0.001). The arrows in (**A**–**G**) indicate the immue-positive cells. For mice grouped into 1–5 the behaviors were tested (n = 12 mice per group). In Mechanical Allodynia test (**H**), ***denotes the significant (P < 0.001) difference between group 1 and other groups, ^###^denotes the significant (P < 0.001) difference between group 1/3/3–24 hrs and group 4/5/5–24 hrs, ^&&&^denotes the significant (P < 0.001) difference between group 1 and group 2/4/5/5–24 hrs. In forced swimming and open field tests, ***denotes the significant (P < 0.001) difference between group 1/3 and 2, ^###^denotes the significant (P < 0.001) difference between group 1/3 and 4, ^&&&^denotes the significant (P < 0.001) difference In the Morris water maze test, *demonstrates the significant difference of time spent in target quadrant compared to other quadrants (*P < 0.05, ***P < 0.001). (**I**) Behavior testes for Mechanical Allodynia, depressive and anxiety (Mean ± S.E.M, n = 6). In Mechanical Allodynia test, ***denotes the significant (P < 0.001) difference between group1 and other groups, ^###^denotes the significant (P < 0.001) difference between group1/group3/group3–24 hrs and group4/group5/group5–24 hrs, ^&&&^denotes the significant (P < 0.001) difference between group1 and group2/group4/group5/group5–24 hrs. In forced swimming and open field tests, ***denotes the significant (P < 0.001) difference between group1/group3 and group2, ^###^denotes the significant (P < 0.001) difference between group1/group3 and group4, ^&&&^denotes the significant (P < 0.001) difference between group1/group3 and group5. (**J**) Behavior changes in the spatial working memory (Mean ± S.E.M, n = 6). In the bar figures, *demonstrates the significant difference of time spent in target quadrant compared to other quadrants (*P < 0.05, ***P < 0.001). The scatter diagram describes the time spent in target quadrant (*P < 0.05, **P < 0.01) and the mean speed (*demonstrates the significant difference between group1/group3 and other groups, P < 0.01)

On one hand, SNL induced BDNF deficiency was only found in anterior cingulate cortex (ACC), CA1 and CA3 of the hippocampus, basolateral amygdala (BLA) and rostroventral medulla (RVM). These deficiencies ranged from D7 to D21 (Fig. 2A-E). On the contrary, the WTD-mediated rescue effect was relative delayed (Fig. 2A-E, Fig. S4). On D7, compared to SNL, WTD mediated up-regulation was limited in RVM; On D14, the remissions by WTD group was extended to RVM; On D21, the up-regulations by WTD were widely detected in ACC, RVM, BLA and CA3. In sum, WTD mediated rescue of BDNF is limited in ACC, CA3, BLA and RVM. On the other hand, for TNF-α, SNL-induced increases of TNF-α expression level were restricted to CA1 and CA3 (Fig. 2F-G). In addition, as compared to SNL, the effects of WTD administration were limited to CA3 (Fig. 2G). In sum, the pathological alternations as well as the pharmacological remissions of BDNF and TNF-α are proven to focus on CA3, which encourage more detailed pharmacological analysis in this region. In view of the WTD mediate co-regulative effects of BDNF and TNF-α in CA3, more elaborate quantifications of these two proteins were performed during the disease progression in CA3 on D1, 3, 7, 9, 14, 21, individually. The results were consistent with the findings above (Fig. S2). As expected, the deficiency of BDNF initiated on D7 and gradually rescued by WTD until D21. Moreover, the increased TNF-α level was from D1, and gradually decreased from the beginning to the end during the whole process of WTD administration (Fig. S2). Considering the specific pathological and pharmacological regulations of both BDNF and TNF-α in CA3, the important roles of these molecules in hippocampus-related pathological progression suggest the crucial effect of WTD on the CA3.

To investigate whether the effects of WTD was restricted to CA3, ANA-12 (selective antagonist of BDNF-TrkB signal pathway), together with TNF-α were injected into CA3 to inhibit the WTD mediated modulations of BDNF and TNF-α. The behavior consequences were assessed by Von Frey filaments test, forced swimming test, the open field test and Morris water maze. Compared to sham ones (group 1), in mice underwent SNL operations (group 2) nociceptive symptoms were shown by decreases in pain threshold (Fig. 2H), depressive symptoms were shown by increases in immobility time in forced swimming test (Fig. 2I), anxiety symptoms were shown by decrease in central duration time in open field test (Fig. 2I) and cognitive symptoms (Fig. 2J) cognitive deficits were shown by the indiscriminate time spent in each quadrant in Morris water maze. In addition, the administration of WTD (group 3) was found co-curative for the above symptoms developed in SNL ones. Based on above results, we confirmed the co-curative effects of WTD in SNL mice. Similar to SNL, in mice underwent the combinative CA3 injections of ANA-12 and TNF-α (group 4), we detected the nociceptive (Fig. 2H), depressive (Fig. 2I), anxiety and cognitive deficits (Fig. 2J). In this way, the combinative modulation of BDNF and TNF-α in CA3 is proven inducible of the neuropathic pain syndromes; For WTD, in spite of the co-curative effects in SNL, no significant remission by the was detected after the administration of WTD in mice under both the SNL and combinative CA3 injection (group 5). The additional combinative injection in CA3 is proven resistant to the co-curative effects of WTD to SNL operations. In summary, the analgesic, emotional and cognitive regulation effects of WTD are modulated by the BDNF-TrkB pathway and the dose of TNF-α in the CA3.

**Figure 3:**
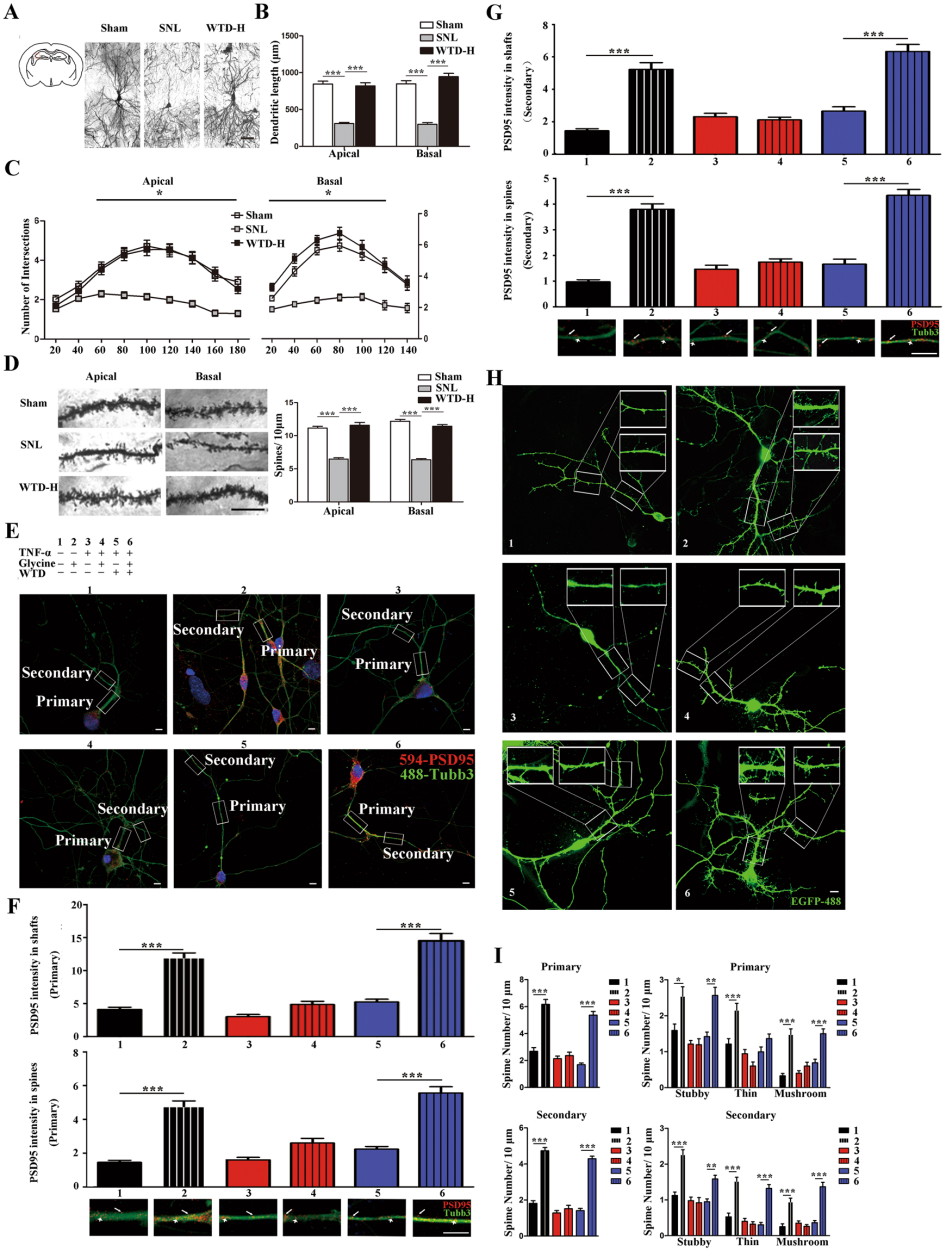
The SNL induced atrophy and WTD mediated rescue of hippocampal CA3 pyramidal neurons on D21. (**A**) – (**D**) presented the atrophy and rescue of CA3 pyramidal neurons on the right side (n = 4 mice per group). (**A**) Shows the location and the imaging of the entire pyramidal neurons, scale bar 100 μm. The atrophy is evaluated from dendritic length (**B**), dendritic intersections (**C**), and spine density (**D**) (enlarged pictures show the spine density, scale bar 10 μm) (*P < 0.05, ***P < 0.001) (Mean ± S.E.M, at least 38 neurons pictured from 3 mice were analyzed in each group). (**E**–**I**) presented the TNF-α mediated inhibition and WTD mediated rescue of primary hippocampal neurons undergo the activation by glycine. (**E**) Indicates the grouping conditions and the Z-projection confocal images of the TUBB3 labelled neurons with primary and secondary dendrites, in which the red dots indicates the positive dyeing of PSD95, scale bar 10 μm). The magnification of the primary (**F**) and secondary (**G**) dendrites, in which the arrowheads indicate the expression of PSD95 in the shafts and the arrows indicate the expression of PSD95 in the spines. Quantification data is shown in bar figures (***P < 0.001, Mean ± S.E.M, at least 30 neurons pictured from 3 cultures are analyzed in each group). (**H**) Z-projection confocal images of the GFP labelled neurons with the enlarged photos showing the stubby, thin and mushroom spines in both the primary and secondary dendrites, scale bar 10 μm. (**I**) quantification data is shown in bar figures (**P < 0.01, ***P < 0.001, Mean ± S.E.M, at least 30 neurons pictured from 3 cultures are analyzed in each group).

### WTD rescued the dendrite morphology and synapse remodeling of hippocampal neurons

Given the significant effect of WTD on the behavioral, we next tested whether there exist alterations of morphology and neuronal activity exposure to the drugs in CA3 pyramidal neurons *in vivo* and explored the pharmacological mechanism in cultured primary hippocampal neurons *in vitro*.

**Figure 4:**
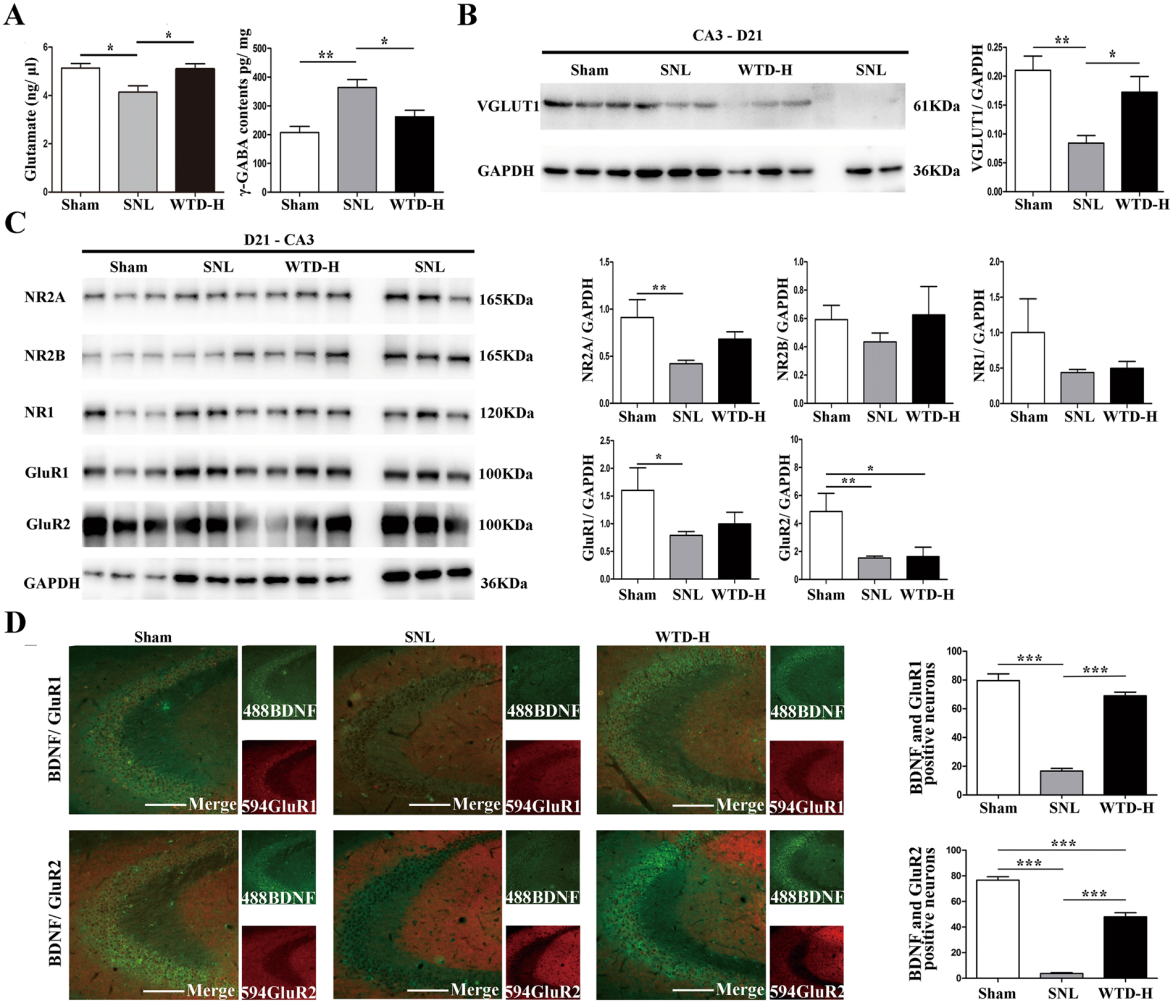
The SNL induced hypofunction and WTD mediated remission of the glutamate system in the hippocampus CA3 region on D21. Tissues on the right side are analyzed (*P < 0.05, **P < 0.01, ***P < 0.001).(**A**)Alternated expression of glutamate and γ-GABA (Mean ± S.E.M, N = 5). (**B**) Altered expression of vGLuT1 (Mean ± S.E.M, 4–5 mice per group), using GAPDH as the loading control. (**C**) Altered expression of NR1, NR2A, NR2B, GluR1 and GluR2 (Mean ± S.E.M, 3 mice analyzed in Sham/WTD group and 6 in SNL), using GAPDH as the loading control. The gel pictures in 4B and C have been cropped and the original full-length gels are presented in supplementary Fig. S3. (**D**) The quantification of BDNF and GluR1/GluR2 co-localization CA3 pyramidal neurons by double immunofluorescence (Mean ± S.E.M, N = 3), scale bar 200 μm.

To achieve these goals, Golgi staining was used to evaluate the neuronal morphology *in vivo*, including the length, intersections of dendrites and the density of dendritic spines both basally and apically. Compared to the sham, the chronic stresses compromised decreases in dendritic length (Figs 3A and 4B), number of intersections (Fig. 3C) and dendritic spine density apically and basally (Fig. 3D) in the SNL mice. In addition, compared to the SNL, administration of WTD significantly rescued the loss of dendritic spines and retraction of dendrites (Fig. 3).

Given the drug-induced changes of the morphology of CA3 neurons, we would evaluate whether WTD interfere with the TNF-α-induced alterations of spine remodeling *in vitro* (n = at least 30 neurons from three cultures). For neurons incubated with PBS, increased the number of stubby, thin, and mushroom spines (Fig. 3H and I), as well as the intensity of PSD95 in both shafts and spine regions (Fig. 3E,F and G) were observed exposure to glycine. These morphological alternations in sum indicate the chemically activation of spines by glycine. On the contrary, for neurons incubated with TNF-α and glycine, no significant increase was observed (Fig. 3E–I), which suggest that TNF-α is effective for the ablation of the glycine-induced synaptic remodeling. Furthermore, in spite of TNF-α, increases of PSD95 and the stubby (in both primary and secondary dendrites), thin (in secondary dendrites) and mushroom (in both primary and secondary dendrites) spines were achieved by glycine in neurons pretreated with WTD (Fig. 3I). As shown with the reversible rescue of TNF-α mediated inhibition, the administration of WTD is proven protective for primary hippocampal neurons. In sum, both *in vivo* and *in vitro*, we demonstrated the WTD-mediated rescue in the loss of hippocampal neurons and synaptic morphological changes.

### WTD rescued the glutamate deficiency in hippocampal CA3 induced by STL

The plasticity of glutamatergic pyramidal neurons is necessary for executing functions of hippocampus. In neuropathic pain and depression, the atrophy as well as the hampered glutamate N-methyl-D-aspartate receptor (NMDAR)-dependent and the α-amino-3-hydroxy-5-methyl-4-isoxazolepropionic acid receptor (AMPAR)-dependent LTP in hippocampus has been widely recognized^31^. In this study, we reported the long-term (detected on D21) morphological regulations in CA3 pyramidal neurons and the rescue of the PSD95 level mediated by WTD, which suggests WTD may be crucial for the activity-dependent transmission of NMDAR and AMPAR and the plasticity of glutamatergic neurons.

To validate whether WTD modify the plasticity of hippocampal neurons in the long term of SNL progression, the quantifications of molecules related to the plasticity of glutamatergic neurons were performed on D21. The excitatory neurotransmitter glutamate level, its pre-synaptic receptor VGLuT1 and post-synaptic submits of NMDAR and AMPAR, as well as the inhibitory neurotransmitter γ-GABA were quantified in the CA3 region (The full-length gels of western-blot are shown in supplementary Fig. S3). Reduced glutamate and enhanced γ-GABA level were observed in SNL compared to sham, which indicates the imbalance between the inhibitory and excitatory systems in CA3. In addition, compared to SNL, increased glutamate and decreased γ-GABA level represented exposure to WTD-H, which indicates the remission of the imbalance between the excitatory and inhibitory systems in CA3 by WTD (Fig. 4A). The expression of VGLuT1 was reduced in SNL group and elevated by WTD-H, which indicates the WTD mediated rescue of the SNL induced hypofunction of CA3 pyramidal neurons (Fig. 4B). Next, western blot analysis of the post-synaptic receptors showed specific reductions in NR2A, GluR1and GluR2 submits in the SNL group, which indicates the hypofunction in SNL is related to both the NMDAR and AMPAR. However, compared to SNL, no significant up-regulation was found by WTD-H (Fig. 4C), which indicate that the effect of WTD was mainly on the pre-synaptic receptors. Furthermore, by double immune staining of BDNF and GluR1/GluR2, we consolidated the SNL induced decreased BDNF/GluR1 double-positive CA3 neurons and identified the partial remission mediated by WTD-H. Selective up-regulation of AMPAR-positive CA3 neurons indicate the WTD-mediated rescue may be dependent on the activation of AMPAR (Fig. 4D). In sum, in the late stage of SNL progression (D21), our findings showed decreased function of glutamatergic neurons manifested by the down-regulations of above molecules. Moreover, the WTD-mediated up-regulations of glutamate, VGLuT1 and AMPAR may further indicate the rescue of the hypofunction of CA3 pyramidal neurons.

### WTD rescued the plasticity deficits of hippocampal CA3 pyramidal neurons in SNL mice

**Figure 5:**
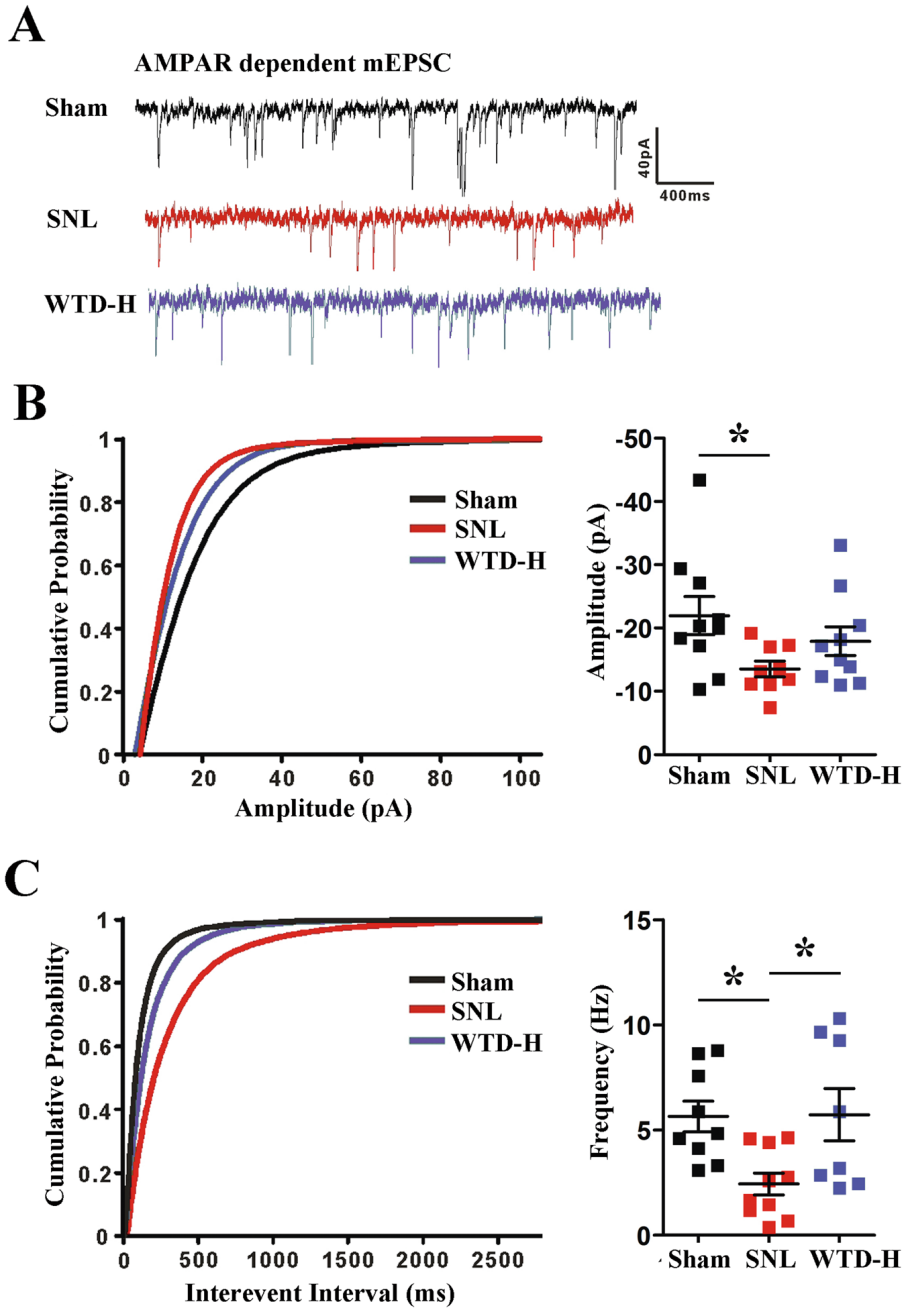
The SNL induced hypofunction and WTD mediated remission of the AMPAR-dependent mEPSC in the hippocampus CA3 pyramidal neurons on D21 (Mean ± S.E.M, n = 9–10 neurons on the right side recorded from 4 mice) (*P < 0.05). (**A**) Representative mEPSCs recorded at a holding potential of −80 mV. (**B**) Cumulative probability plot and averages for mEPSC amplitudes. (**C**) Cumulative interevent interval plot and averages for mEPSC amplitudes.

To further reveal the underling molecular and morphological alternations and further explore the mechanisms of WTD on the CA3 neurons from the electrophysiology perspective, we performed an AMPAR-dependent mEPSC analysis on D21 (Fig. 5A).

Compared to the sham group, a cumulative probability analysis showed decreased mEPSC amplitude and interevent interval in the CA3 pyramidal neurons of the SNL mice (Fig. 5B and C), which indicate SNL-induced alterations of both pre-synaptic and post-synaptic CA3 neurons. In addition, compared to SNL, selective improvements of the frequency were found in the WTD-H group (Fig. 5C). Whereas, no significant improvement of amplitude was found with WTD administration (n = 10 neurons from 3 mice) (Fig. 5B). In summary, we concluded that the WTD-mediated rescue of the AMPAR-dependent plasticity of hippocampal CA3 pyramidal neurons is more likely associated with the pre-synaptic mechanisms.

## Discussion

### WTD shows curative effects to neuropathic pain in the central nervous system

The difference between inflammatory and neuropathic pain exist in the maladaptive alternations of neurons in the central nerves system (CNS)^14^. In inflammatory pain, the hyper-activation of the central nerves system is dependent on the peripheral neurons. The ablation of the nociceptive signals by either the resolution of initial injures or the inhibition of nociceptive receptors has been proven effective^32^. However, for NP, the heightened sensitivity of neurons are evoked but independent of the initial injuries, which make the simple curation of peripheral injures ineffective. Therefore, the effective controlling of pain in NP is dependent on the remission of the over-activated afferent neurons located in CNS. In addition, for NP, the effective remission of pain-induced psychiatric disorders is as important as the controlling of nociceptive symptoms^33^. Therefore, drugs for NP should further focus on brain nuclei which was crucial for the affective aspects of pain.

WTD has been widely applied in inflammatory pain. In spite of the relative clear mechanism validated in the inhibition of the peripheral nociceptive receptors and pro-inflammatory factors, the central effects and mechanism of WTD has never been reported. In this study, SNL mice were used for two reasons. Firstly, compared to other models, the individual difference in nociceptive symptoms among SNL mice is smaller. Secondly, multiple studies confirmed the accompanied affective consequences in SNL mice. On the contrary, in PSNL, CCI and SNI models, the induction of affective symptoms is uncertain and dependent on the specific conditions^4^. In addition, the first line drug pregabalin, which shows favorable analgesic but not anti-depression effects^7^, was used as the positive control.

Firstly, the specificity of WTD was represented. We validate that in shamed mice the pain threshold were undisturbed by long-term application of high dose WTD (for 21 consecutive days) (see Supplementary Fig. S4), suggesting that the WTD mediated modulation is limited to the hypersensitivity conditions and do not influence the normal response to nociceptive stimulus. Secondly, the analgesic effects of WTD are more long-lasting compared to pregabalin. In spite of the insignificant fluctuations with the prolongation of day, we observed gradual increases of pain threshold in the efficacy of WTD. On the contrary, with the prolongation of administration, the analgesic effects of PGB detected 24 hrs after administration were dwindling and disappeared on D11. In addition, the sleepiness brought by PGB is serious. Thus, WTD may facilitate to reduce to drug tolerance in the clinical treatment. Thirdly, as shown by the increases of central duration time in open field test and decreases in the immobility time in forced swimming test, WTD was effective for the restoration of depressive and anxiety behaviors. As detected on D7, D14 and D21, the anti-depression and anxiety effects of WTD are long-lasting. The 21-day continuous administration does not induce the tolerance to WTD. For the first time, we explored the stable analgesic as well as the anti-depression and anxiety effects of WTD for neuropathic pain. The co-curative effects definitely indicate the central mechanism of WTD, which has not been explored before.

### WTD sub-serve the co-curative effects by regulating the level of BDNF and TNF-α in CA3

For NP, several brain regions such as thalamic nuclei, primary and secondary somatosensory cortex, insula, anterior cingulate cortex, posterior parietal cortex, amygdala, hippocampus, rostroventral medulla and PAG have been proven relevant to the nociceptive, affective and cognitive procession of pain stimuli^34–36^. However, in this study, we aim to figure out the direct target of WTD.

Firstly, by the quantification of BDNF, we consolidate the extensive mal-regulation of BDNF in SNL and the rescues by WTD, which may scope both the direct and indirect drug targets. Secondly, we aimed to figure out which brain nuclei can explain for, but not merely relevant to the comorbidity in neuropathic pain, as well as the co-curative effects of WTD. For this aim, we analyzed the expression of TNF-α in brain regions underwent the pathological and pharmacological modulations of BDNF. We found that, different from BDNF, the SNL induced up-regulation of TNF-α is found but limited in hippocampus (CA1 and CA3). Further, the selective remission of TNF-α, mediated by long-term administration of WTD, was found and limited in CA3. Similar to BDNF, TNF-α is crucial for the plasticity of neurons. Especially, the hippocampal injections of TNF-α has been related with the mal-regulation of AMPAR/NMDAR and further the impairment of the plasticity of hippocampal glutamatergic pyramidal neurons and accounts for the nociceptive and cognitive symptoms^17,29,30^. In addition, in NP, the mal-regulation of BDNF, induction of the atrophy of hippocampus and the depressive behaviors has been proven dependent on TNFR1^18,27^. In sum, unlike BDNF which is widely regulated in multiple brain regions and responsible for both the direct and indirect consequence of NP, the specific modulation of TNF-α in hippocampus may be directly associated with the comorbidity. Indeed, more studies are needed to clarify the pathological mechanism leading to the specific up-regulation of TNF-α in hippocampus and to elucidate the signal pathways associated with the up-regulated TNF-α and the subsequent behavioral consequences. Especially, with the clarification of whether the pathological alternation of BDNF in hippocampus is modulated by TNF-α or not will give more light on the mechanism of TNF-α and the explanation of the roles hippocampus assumed in the comorbidity.

Further, we explored whether CA3 alone can explain for the comorbidity in NP and the co-curative effects of WTD. By the stereotactic injection, TNF-α was up-regulated and the BDNF-TrkB signal pathway was inhibited in CA3. By the behavioral tests, we found that the stereotactic injection alone is enough to induce the nociceptive, affective and cognitive symptoms, which suggest the important roles CA3 assumed in the comorbidity. In addition, in SNL mice, the co-curative effects of WTD were totally ablated by the injection, which further suggest CA3 as the specific target of WTD. For the first time, hippocampal CA3 is confirmed as the basis of comorbidity and encourage more studies in this region.

### WTD rescue the plasticity of glutamatergic pyramidal neurons of CA3

So far, whether the plasticity of CA3 pyramidal neurons alternated in SNL is still unknown. In neuropathic pain, studies concerning the morphological alternations have been limited in CA1^37^. However, for depression, the stress-induced atrophy was reported both in CA1 and CA3. In our study, by analyzing the dendritic branching, length and spine density of CA3 neurons we validated the SNL induced pathological atrophy and WTD mediated rescue of CA3, as well as in CA1. These findings provide more evidence that pain and associated depression symptom may share the same mechanism. CA3 may underling both the nociceptive and affective consequences of neuropathic pain. Thus, the clarification of the plasticity of CA3 neurons indeed helps for the understanding of the mechanism of co-morbidity.

In cultured primary hippocampal neurons, we found that TNF-α negatively affected the activity-dependent turnover of spines by glycine, and WTD successfully restore the inhibition of TNF-a. Moreover, PSD95, the activation of which by glycine was proven inhibited by TNF-α and restored by WTD. The effective regulation of PSD95 further indicates that the WTD-mediated rescue is possible dependent on NMDAR or AMPAR. Prior studies have reported PSD95 as the role of NMDAR activation and mediating the AMPAR targeting, as well as other excitatory transmissions in the CNS^38^. Therefore, we conclude that TNF-α is harmful to the plasticity of glutamatergic neurons. By the regulating of AMPAR and NMDAR, the administration of WTD maybe focused on the plasticity of CA3 glutamatergic neurons.

In SNL mice, we consolidate our hypothesis. In CA3, the pathological down-regulation of glutamate and VGLuT1 were confirmed and rescued by WTD, which further indicates the CA3 glutamatergic neurons as the drug target. Even though prior studies have reported the dysfunction of glutamatergic neurons, different patterns appeared in our study. For example, in depression, the reduced expression of AMPAR has been widely reported and its restoration was considered to be the crucial indicator of treatment benefits^39^. In NP, the NMDAR-dependent alternations (not AMPAR) of CA1 neurons were more likely to response with the up-regulation of TNF-α and contribute to the induction of cognitive symptoms^40^. In this study, different from the reported down-regulation of NMDAR submits (NR2B NR1) in NP^31,41^, we reported the down-regulation of NMDAR NR2A submit, pre-synaptic VGLuT1R, AMPAR GluR1 and GluR2 submits in CA3. In addition, the rescue of AMPAR shown by the increases of AMPAR positive neurons in CA3 by WTD further indicate the participations of AMPARs in the comorbidity.

In this study, more emphasis were focused on the comorbidity between pain and depression. For this aim, prior to the quantitation, animals were underwent the behavior tests. We found that approximately 10% of mice underwent SNL operations did not development the serious nociceptive symptoms. In addition, approximately 30% mice with serious nociceptive behaviors did not develop the depressive or anxiety symptoms. In sum, the discrepancy of the depressive or anxiety symptoms may occur among the studies, dependent on feeding and detection conditions. In this study, under the strict behavior screening, we reported the important roles CA3 and the AMPARs assumed in the comorbidity. Finally, AMPAR dependent mEPSC analysis manifested the SNL induced and WTD rescued hypofunction of CA3 glutamatergic neurons. These pre-synaptic recue effects of WTD, indicated by the specific improvements on the mEPSC frequency, explain the remission of glutamate deprivation and up-regulation of the pre-synaptic receptor VGLuT1.

## Materials and Methods

### Animals

Adult (8 week, 26–28 g) male ICR (abbreviation of the Institute of Cancer Research) mice were kept under a 12hr-light-dark cycle, with available food and water. After a 7-day habituation, all animals were tested for the baseline mechanical allodynia, depression and anxiety. Those with normal behaviors were randomly divided into experimental groups. Adequate measures were taken to minimize pain or discomfort.

### The Spinal Nerve Ligation

Spinal nerve ligation mice, with obvious pain and psychiatric disorders and no significant locomotor deficit, is widely accepted in the studies of neuropathic pain^42^ were constructed as reported^43^. Briefly, the mice were anesthetized with isoflurane inhalation anesthesia. The L5 spine nerve on the left side was exposed and tightly ligated with a surgical suture. The skin was then washed with normal saline and sutured. Sham procedures were conducted in the same manner, with the naked L5 spine nerved un-ligated.

### Drugs

Pregabalin (Pfizer, J20100102), was selected as positive control (dissolved in distilled water to 75 mg/ml, administered 0.3 ml/30 g body weight); According to the quality control standards of the China Pharmacopoeia, WTD is consisted of five herbs (Radix Aconiti/Wu Tou, Herba Ephedrae/Ma Huang, Radix Astragali/Huang Qi, Raidix Paeoniae Alba/Bai Shao and Radix Glycytthizae/Gan Cao). By UPLC-Q-TOF-MS, the major constitutions of WTD were further clarified^10,44–46^. For the water extract of WTD, more detailed standards such as the quantification of eleven main components with specific anti-inflammation effects (Benzoylmesaconine, Aconitine, Benzoylhypacoitine, Benzoylaconitine, Hypaconitine, Ephedrine, Calycosin-7-glucoside, Glycyrrhizic acid, Liquiritin, Formononetin, Liquiritigenin) have been used as quality monitoring in our studies^12^. Three doses described as high (0.3 ml/30 g body weight, equivalent to 2 times of the dose clinically prescribed for rheumatoid arthritis patients), medium (0.15 ml/30 g body weight) and low (0.075 ml/30 g body weight) were administered. In the Sham and SNL groups, WTD was replaced with an equal volume of distilled water. All drugs and distilled water were administered by gavage at 7:00 am, from D1 to D21 after SNL or Sham operation.

The drug for stereotactic injections was a mixture of TNF-α and ANA-12. Recombinant mouse TNF-α (ab157351) was dissolved in normal saline and stored at the concentration of 20 μg/ml as the storage solution. 10 mM ANA-12 (MCE HY-12497) were diluted with saline at the ratio of 1:1000 and stored as the storage solution. Before the usage, the working solution is made by 1 μl of the stored TNF-α and 1 μl of stored ANA-12 and 98 μl normal saline.

### Mechanical Allodynia

From D0 to D21 after the operations, the von Frey filaments tests were performed 1hr and 24 hrs after the daily administration according to previous studies^47^. Briefly, after a 30 min acclimation to the separated cubicles, a Von Frey filaments (0.008–4.0 g) test was conducted for each mouse 6 times, in 5 min intervals. The von Frey filaments were perpendicularly presented to the left paw, held for 5–8 s, with a slight bend of the filament. A positive response is defined as the appearance of an abrupt withdrawal of the paw or by a flinching behavior.

### Sucrose Consumption

On D0/7/14/21 after the operations, sucrose consumption tests were performed^48^ 4 hrs after gavage administration. Briefly, 24 hrs before the test, mice were trained with the test bottle containing 1% sucrose for 12 hrs, then deprived of the water for 12 hrs and acclimated to the test room for 1 hr. The test lasted for 1hr, and the consumption of sucrose water was weighed for each mouse.

### Forced Swimming Test

On D0/7/14/21 or D0/7/11/14 after the operations, a forced swimming test were performed^49,50^ 4 hrs after gavage administration.Briefly, the apparatus of the forced swimming test was a glass cylinder 26 cm in height and 16 cm in diameter containing 14 cm of water at 25 ± 1 °C. Before the test, the mice were acclimated to the test room for 1hr. In the 6 min test, the first 2 min is the adaption stage. In the following 4 min, the duration of immobility and passive swimming is measured.

### The Open Field Test

On D0/7/14/21 or D0/7/11/14 after the SNL or sham operation, the open field test was performed 4 hrs after gavage administration. Briefly, a square white test cage 50 * 50 cm, with a wall 30 cm high and a border region 8 cm wide was used. Before the test, the mice were acclimated to the test room for 1 hr. At the beginning, each one was placed in the border region facing the wall. During the 4 min test time, the total time mice stayed in the central region was measured.

### Morris Water Maze

Spatial working memory was assessed with the water maze as described by Morris^51^. Four virtual quadrants: quadrant 1 (Q1), quadrant 2(Q2), quadrant 3 (Q3) and quadrant 4 (Q4) were divided in the pool (1.2 m in diameter). Water was opacified with black ink and maintained between 20 ± 0.5 °C. In the training stage, mice were trained to locate a hidden platform (10 cm in diameter, submerged 1 cm) in Q3 for 3 successive days, comprising 4 trials/day. In each trial, the mice were released facing the wall and allowed to search the platform for 90 s. Mice that failed to find the platform within this time were guided to the platform and maintained on the platform for 15 s. On the final trial, mice that still failed to find the platform were excluded. On the 4^th^ day, a detection test without the platform was conducted. Each mouse was released facing the wall of Q1 and given 60 s. On the 5^th^ day, 4 trials with the platform placed in Q1 were conducted. On the 6^th^ day, another detection test without the platform was conducted. Each mouse was released facing the wall of Q3 and given 60 s. Recorded with an Ethovision XT monitor, the escape latency was recorded in each trial; the time spent in each quadrant and the mean speed were recorded for the detection day.

### Stereotactic Injections

Firstly, the micro drug delivery trocars were fixed at AP: −1.6 mm, R: −2 mm, DV: −2.1 mm relative to bregma and dural surfaces. One week later, the mechanical allodynia were analyzed. Mice without significant changes were randomly divided into shamed ones (group 1), mice underwent SNL operations without (group 2) and with the administration of WTD (group 3), mice underwent the combinative CA3 injections of ANA-12 and TNF-α (group 4), mice under both the SNL and combinative CA3 injection and the administration of WTD in mice (group 5) (n = 12). From D1 to D7, 2 μl of the working solution containing 1 ng TNF-α and 0.1 pmol ANA-12 were injected for 7 successive days as recommended in the previous studies^17,40,52^.

### Isolation and Culture of Primary Hippocampal Neurons

E18 pregnant ICR mice were sacrificed and the hippocampus were dissected in cold phosphate-buffered saline (PBS).The following procedures were similar to our previous studies^53^. Neurons were plated on the glass covers (Fisher FIS 12-545-82) in the 24-well plates, keeping the concentration at 1 × 10^4^ cells/well.

### Spine Assays

On DIV 12, the primary hippocampal neurons were transfected with plasmids of PEGFP-N1 (Genebank U55762). 0.8 μg PEGFP-N1 were transfected into each 24-well plates as recommended by the protocol of Lipofectamine 2000 (Invitrogen USA). 24 hours later, WTD (final concentration is 5 μg/ml) or equivalent volume of PBS was added; 30 minutes later, TNF-α (final concentration is 5 ng/ml) or equivalent volume of PBS was added; 20 minutes later, Glycine (final concentration is 200 μM) or equivalent volume of PBS was added and incubated for another 40 minutes. Then neurons were immuno-stained with primary antibody to EGFP (Earthox E022410 1:200) and secondary antibody (delight Goat anti mouse 488 earthox E032210 1:400) as recommended in published studies^54^. Z- series Confocal images were obtained with the Olympus FV1000 confocal microscope. Pictures were taken under 100x oil-immersion objective. The stubby, thin and mushroom spines were distinguished as published studies^55^.

### Immuno-staining and Analysis

The double staining of PSD95 and TuBB3 was conducted as follows. Neurons were incubated with primary antibodies (TuBB3, 1:600, Abcam ab78078 USA and PSD95, 1:400, Abcam ab76115 USA) at 4 °C overnight. For secondary antibody, delight Goat anti mouse 488 (earthox E032210 1:400), and Goat anti rabbit 594 (earthox E032420 1:400) were used. Z- series Confocal images were obtained with the Olympus FV1000 confocal microscope. Pictures were taken under 100x oil-immersion objective. In TuBB3 positive neurons, the intensity of PSD95 were analyzed.

### Enzyme Linked Immunosorbent Assay (ELISA)

Whole proteins were extracted from the brain nucleus and quantified to 100 μg/samples. The amounts of BDNF, TNFα, γ-GABA were detected by an ELISA assay (R&D USA) following the manufacture’s protocol and measured at 450 nm.

### Glutamate Assays

Hippocampal CA3 proteins were extracted and diluted to 10 μg/μl. Assays were conducted (Sigma MAK004) following the manufacture’s protocol and measured at 450 nm.

### Diaminobenidime (DAB) Staining

The brain slices were incubated with primary antibody(goat anti-BDNF 1:150 abcam ab75040) at 4 °C overnight. For secondary antibody, the goat and rabbit two-step IHC reagent kits (Goldenbridge Beijing China) were used. The DAB were mixed before usage at 1:20 and incubated for 1–2 min.

### Immuno-staining

The brain slices were incubated with primary antibody(goat anti-BDNF 1:150 abcam ab75040, rabbit anti AMPAR1 1:100 abcam ab183797, rabbit anti AMPAR2 1:600 abcam ab206293) at 4 °C overnight. For secondary antibody, delight donkey anti goat 488(earthox E032231 1:200), and donkey anti rabbit 594(earthox E032421 1:200) were used.

### Western Blot

The primary antibodies used were anti-GAPDH (1:2000 abcam ab8245), anti-NR2A(1:1000 abcam ab124913), anti-NR2B(1:2000 abcam ab81271), anti-NR1(1:2000 abcam ab109182), anti-VGLUT1(1:2000 abcam ab180188). The secondary antibodies used were anti-mouse IgG HRP(1:5000 promega W4208) and anti-rabbit IgG HRP(1:5000 promega W4011). The brands were detected with ECL Western Blotting Substrate (promega w1001).

### Golgi-staining

The adult brains were removed and prepared according to the protocol (FD Rapid Golgistain kit). Serial coronal sections (200 μm) were prepared (Leica VT1200 vibratome, Germany). After the staining, pyramidal neurons in the hippocampal CA3 region were photographed by three-dimensional reconstruction confocal fluorescence microscopy (Olympus FV1000 MPE) at ×400 and ×1000. NeuroJ (plugin of ImageJ NIH) and Sholl analyses were performed to measure the dendritic length, the number of intersections and the density of dendritic spines.

### AMPAR-dependent mEPSC

Acute brain slices were prepared from the hippocampus on the right side and transferred into ice-cooled oxygenated ACSF. Coronal slices with 300 μm thickness were prepared with a vibratome (Leica, VT1000s, Germany). CA3 pyramidal neurons were identified by infrared gradient contrast vedio microscopy. The recordings of mEPSC were performed in whole cell mode. The signal was obtained at a holding potential of −80 mV. For the pharmacological isolation of AMPAR mediated mEPSC, 10 nM glycine, 10 μM bicuculline and D-AP-5 were added to the ACSF. The intracellular solution contained 135 mM K-gluconate, 10 mM HEPES, 2 mM MgCL_2_, 10 mM EGTA, 0.3 mM MgGTP, 0.5 mM NA_2_ATP (pH 7.3 with KOH). The whole extracellular solution contained 0.5 μM TTX. For each cell, a 4 min recording was obtained. For the detection of spontaneous events, the ‘threshold research’ option was used and checked.

### Statistical analyses

For all quantification analyses, one-way ANOVA analyses were used, followed by Tukey post tests. Statistical significance was set at P < 0.05. All of the data in text are presented as the mean ± s.e.m. The sample sizes were noted in the results or figure captions.

### Availability of materials and data

The datasets generated during and/or analysed during the current study are available from the corresponding author on reasonable request.

### Data availability statement

All the primary data supporting this article is available and uploaded with the manuscript.

### Ethical approval and informed consent

All animal experiments were supervised and approved by the Research Ethics Committee of China Academy of ChinThe behavior deficits in SNL mice and the revivalese Medical Science, Beijing, China (permission number: 2016-004) and conducted by the trained researchers following the standards in accordance with NIH guidelines. To ensure the credibility of the experiments, all quantification tests were performed from behaviorally tested ones.

## Electronic supplementary material

Supplementary figure

Dataset 1

Dataset 2

Dataset 3

Dataset 4

Dataset 5

Dataset 6
