# Supplementary material for: Evidence that CA3 is Underling the Comorbidity Between Pain and Depression and the Co-curation by Wu-Tou decoction in Neuropathic Pain
Source: Sci Rep. 2017 Sep 20;7:11935. doi: 10.1038/s41598-017-12184-y (PMC5607326; doi:10.1038/s41598-017-12184-y)
Supplement: Supplementary file 1 — Supplementary figure [file 41598_2017_12184_MOESM1_ESM.pdf]

Title: Evidence that CA3 is Underling the Comorbidity Between Pain and Depression and the Co-curation by Wu-Tou decoction in Neuropathic Pain

Authors: Chunyan Zhu[1#], Qionghong Xu[1], Chao Wang[1], Zhiyun Mao[1], Na Lin[1*]

Affiliation: 1. Institute of Chinese Materia Medica, China Academy of Chinese Medical Sciences, Beijing 100700, China;

[#] **First author:** Chunyan Zhu

* **Corresponding author:** Prof. Na Lin

Address correspondence to: Institute of Chinese Materia Medica, China Academy of Chinese Medical Sciences, No. 16, Nanxiaojie, Dongzhimennei, Beijing 100700, China.

E-mail: linna888@163.com

Phone: +861064014411-2869 **Fax:** +861064013996

Supplementary Fig. S1

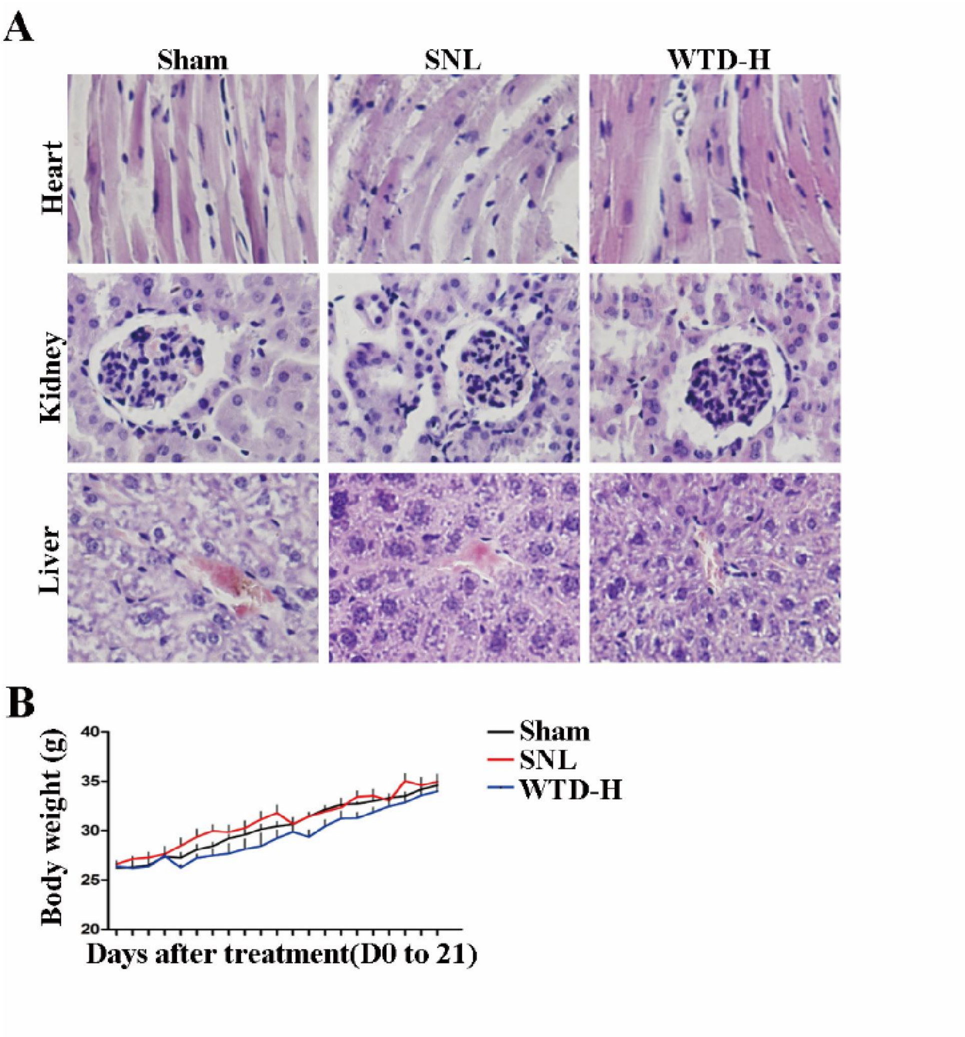

Supplementary Figure 1. The administration of WTD showed no significant injury (A)HE staining on D21 for heart, kidney and liver in Sham, SNL and WTD-H groups. (B) The track record of the body weight from D1 to D21 in Sham, SNL and WTD-H groups.

Supplementary Fig. S2

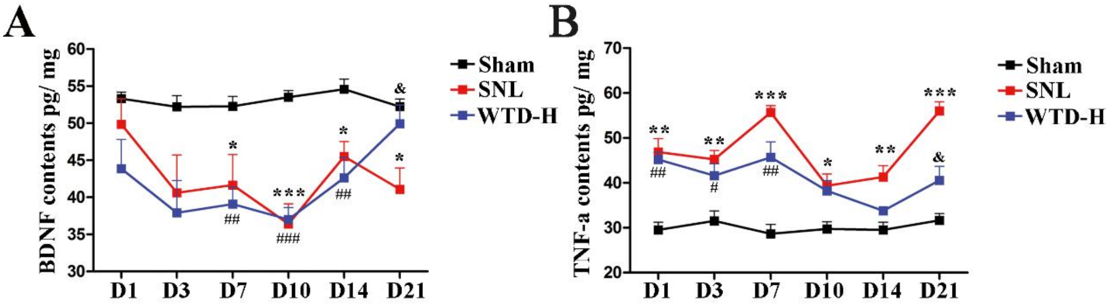

Supplementary Figure 2. The ELISA quantification of BDNF(A) and TNF-a (B) in Sham, SNL and WTD-H groups(Mean ± SEM, at least 6 mice were analyzed in each

group), in which *denotes the significant difference between Sham and SNL (*P < 0.05, ** P < 0.01, ***P < 0.001), $^{\#}$denotes the significant ($^{\#}$P < 0.05, $^{\#\#}$P < 0.01, $^{\#\#\#}$P < 0.001), $^{\&}$denotes the significant($^{\&}$P < 0.05, $^{\&\&}$P < 0.01, $^{\&\&\&}$P < 0.001)difference between WTD-H and SNL.

Supplementary Fig. S3

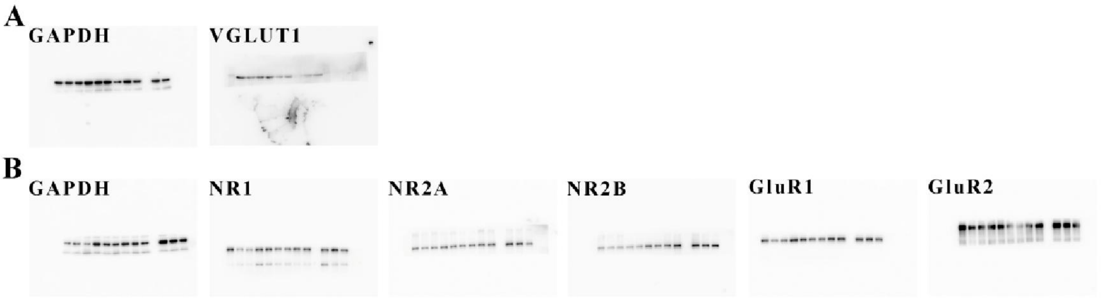

Supplementary Figure 3. The original full-length figures for gels shown in Fig. 4B(A) and 4C(B).

Supplementary Fig. S4

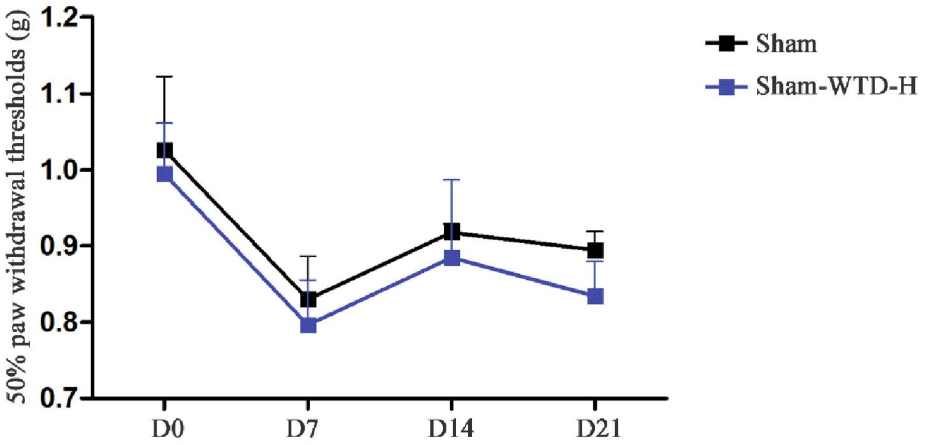

Supplementary Figure 4. The specificity of WTD. Grouped data (Mean ± S.E.M, n=8 per group) show no difference in pain thresholds between Sham and Shamed ones administrated with WTD-H for 21days.
